# Impact of GeneXpert MTB/RIF® on treatment initiation and outcomes of RIF-resistant and RIF-susceptible TB patients in Vladimir TB dispensary, Russia

**DOI:** 10.1186/s12879-020-05243-9

**Published:** 2020-07-25

**Authors:** Julia V. Ershova, Grigory V. Volchenkov, Tatiana R. Somova, Tatiana A. Kuznetsova, Natalia V. Kaunetis, Dorothy Kaminski, Olga V. Demikhova, Larisa N. Chernousova, Irina A. Vasilyeva, Eleanor M. Kerr, J. Peter Cegielski, Ekaterina V. Kurbatova

**Affiliations:** 1 U.S. Centers for Disease Control and Prevention, Atlanta, GA USA; 2 Vladimir Oblast TB Dispensary, Vladimir, Russia; 3 Central Tuberculosis Research Institute, Moscow, Russia; 4 Emory University, Atlanta, GA USA

**Keywords:** Drug resistance, Rifampicin, GeneXpert MTB/RIF

## Abstract

**Background:**

The main advantage of GeneXpert MTB/RIF**®** (Xpert) molecular diagnostic technology is the rapid detection of M*.tuberculosis* DNA and mutations associated with rifampicin (RIF) resistance for timely initiation of appropriate treatment and, consequently, preventing further transmission of the disease. We assessed time to treatment initiation and treatment outcomes of RIF-resistant and RIF-susceptible TB patients diagnosed and treated in Vladimir TB Dispensary, Russia in 2012, before and after implementation of GeneXpert MTB/RIF® diagnostic technology.

**Methods:**

All adult patients suspected of having TB during February–December 2012 underwent a clinical examination, chest x-ray, microscopy, culture, and phenotypic drug susceptibility testing (DST). Starting August 2012 Xpert diagnostic technology became available in the facility. We used logistic regression to compare treatment outcomes in pre-Xpert and post-Xpert periods. Kaplan-Meier curves and log-rank test were used to compare the time to treatment initiation between the groups.

**Results:**

Of 402 patients screened for TB during February–December 2012, 338 were diagnosed with TB (280 RIF-susceptible, 58 RIF-resistant). RIF-resistant patients in the post-Xpert group started treatment with second-line drugs (SLD) earlier than those in pre-Xpert group (median 11 vs. 37 days, Log-rank *p* = 0.02). The hazard ratio for time to SLD treatment initiation was significantly higher in post-Xpert group (HR:2.06; 95%CI:1.09,3.89) compared to pre-Xpert group. Among the 53/58 RIF-resistant TB patients with available treatment outcome, 28 (53%) had successful outcomes (cured/completed treatment) including 15/26 (58%) in post-Xpert group versus 13/27 (48%) in pre-Xpert group. The observed difference, however, was not statistically significant (OR:0.69; 95%CI:0.23,2.06).

Among RIF-susceptible TB cases time to treatment initiation was not significantly different between the groups (2 vs. 3 days, Log-rank *p* = 0.73). Of 252/280 RIF-susceptible TB cases with treatment outcome, 199 (79%) cases had successful outcome including 94/114 (82%) in post-Xpert group versus 105/138 (76%) in pre-Xpert group (OR:0.68; 95%CI:0.36,1.26).

**Conclusion:**

We observed that availability of Xpert for initial diagnosis significantly reduced the time to SLD treatment for RIF-resistant patients in the Vladimir TB Dispensary. Although implementation of rapid diagnostics did not improve treatment outcomes, early diagnosis of MDR-TB is important for selection of appropriate treatment regimen and prevention of transmission of drug-resistant strains of TB.

## Background

With over 10.0 million incident cases and more than 1.4 million death in 2018, tuberculosis (TB) remains a major threat to global public health [1]*.* The burden of drug-resistance is of main interest and concern at global, regional and country levels; emergence of drug-resistance leads to increased morbidity, mortality and healthcare costs [1]*.* Timely and accurate diagnosis of drug-resistant TB, including multidrug-resistant TB (MDR-TB, TB that is resistant to at least rifampicin (RIF) and isoniazid) and initiation of treatment with second-line anti-TB drugs (SLD) is crucial to reduce illness and death and halt disease transmission.

Compared to conventional diagnostic algorithms, use of the GeneXpert MTB/RIF® (Xpert) technology has been shown to be cost-effective for diagnosis among presumed TB patients, drastically decrease the time to diagnose patients in clinical settings, and allow quicker initiation of SLD treatment for MDR-TB [1–4]. A recent meta-analysis based on data from 95 studies was consistent with the findings reported previously, namely that Xpert MTB/RIF has high sensitivity and specificity for diagnosing pulmonary TB and rifampicin resistance [5]*.* For this reason, the World Health Organization (WHO) recommends implementation of Xpert for all patients with presumptive MDR-TB, particularly in countries with high rates of MDR-TB, such as Russia [1, 6].

The impact of Xpert testing on patient outcomes varies widely by country and setting, indicating that Xpert implementation needs to be studied on a place-by-place basis [1]. In 2012, Russia was ranked as having the third highest burden of MDR-TB worldwide, with 45,000 estimated MDR-TB cases among notified pulmonary TB cases [7]. The coverage of drug susceptibility testing (DST) in Russia was incomplete and varied across the regions [7–9].

We evaluated the impact of Xpert rapid molecular testing on patient treatment outcomes and time for RIF-resistant TB patients to begin SLD treatment in Vladimir Region, Russia, at the initial stage of implementation of the technology in the region.

## Methods

Using prospective cohort design, we assessed impact of Xpert on treatment initiation and outcomes of RIF-resistant and RIF-susceptible TB patients in Vladimir Regional TB Dispensary (the Dispensary), Russia. The Dispensary is a referral center for TB patients in Vladimir region and serves about 25% of all TB patients in the region [10]. The patients were referred to the Dispensary as presumptive TB patients after visiting primary health care physician or pulmonologist based on symptoms or results of mass TB screening.

We enrolled all eligible consecutive adults (18 years old and above) with high clinical suspicion of TB accessing care in the Dispensary during February – December 2012. High clinical suspicion of TB was based on clinical symptoms (e.g. chronic cough that lasts 3 weeks or longer, hemoptysis, pain in the chest, fever, weight loss, poor appetite, weakness or fatigue, sweating at night), medical history (e.g. having diabetes, HIV infection, substance abuse or other immunosuppressive condition), social history (e.g. history of imprisonment, homelessness, being healthcare or migrant worker), being close contact of a person with infectious TB disease, and/or chest x-ray findings.

All patients underwent a clinical examination, radiology and bacteriology testing including smear, phenotypic culture and drug susceptibility testing (DST) on Lowenstein- Jensen (LJ) media and using Bactec Mycobacteria Growth Indicator Tube 960® (MGIT) according to national diagnostic guidelines. The facility began implementation of molecular technology for TB diagnosis in August 2012 as “adds-on” test. Performance of Xpert compared to conventional methods in the Vladimir TB Dispensary has been described previously [11]. We defined RIF resistance as resistance detected by any one of LJ, MGIT, or Xpert and used as a proxy marker for MDR-TB. We used the WHO definition and considered cure or treatment completion as successful outcomes; treatment failure, loss to follow up or death were considered poor outcomes [12].

We used Kaplan-Meier curves to describe the time to initiation of treatment with SLD among RIF-resistant TB patients tested before (pre-Xpert) and after Xpert implementation (post-Xpert), and the log-rank test to assess homogeneity between the groups. We restricted survival analysis to the first 60 days after initial diagnosis because LJ DST results were available within 60 days. We censored patients who never started SLD treatment during this period, died or were lost to follow-up before starting SLD treatment. We used Chi-square test to compare median time to treatment initiation between the groups and Cox proportional hazard modeling to measure the association between availability of Xpert for initial diagnosis and probability of SLD treatment initiation.

We assessed impact of Xpert and patient characteristics on treatment outcomes of RIF-resistant and RIF-susceptible TB using logistic regression and mid-p approach. Analyses were conducted utilizing SAS software version 9.3 (Cary, NC).

## Results

**Fig. 1:**
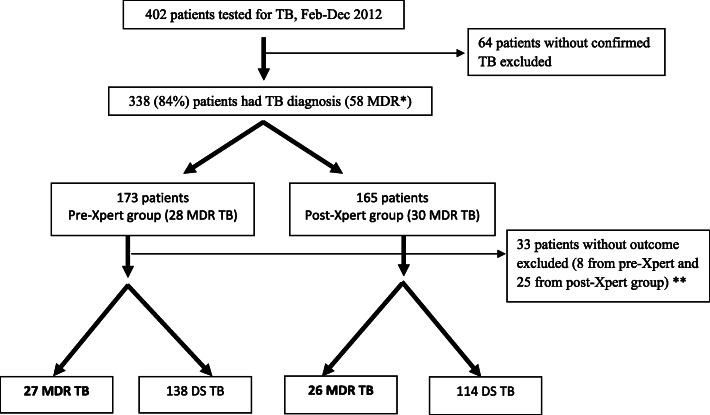
Participant flow chart. * 53/58 RIF-resistant patients were included in Kaplan-Meier and Cox Proportional Hazard analysis (28 from pre- and 25 from post-Xpert group); 5/30 RIF-resistant patients from Post-Xpert group did not have dates of SLD treatment initiation; ** 305 patients were included in treatment outcome analysis (252 RIF-susceptible and 53 RIF-resistant patients)

Sputum specimens from 402 enrolled individuals with suspected TB were tested for M. tuberculosis between February–December 2012 in the Vladimir TB Dispensary. Of 402 enrolled patients, 338 were diagnosed with TB, including 173 (51%) in pre-Xpert group and 165 (49%) in pos-Xpert group (Fig. 1). Characteristics of patients enrolled in each group are presented in Table 1. Among 338 enrolled TB patients, 58 (17%) had RIF-resistant TB including 28 in the pre-Xpert group and 30 in post-Xpert group; 90% (305/338) had data on treatment outcome. DST results to FLD and SLD for RIF-resistant TB patients are provided in Table 2; for 8 patients from post-Xpert group DST results were not available for analysis. However, patients with RIF resistance identified by Xpert were considered MDR and received SLD treatment.

**Table 1:** Sociodemographic and clinical characteristics of patients in pre-Xpert and post-Xpert group

Characteristics		Pre-Xpert ***N*** = 173	Post-Xpert ***N*** = 165	p *
**Age**				0.8
	**15–24**	19 (11%)	15 (9%)	
	**25–34**	49 (28%)	49 (30%)	
	**35–44**	31 (18%)	35 (21%)	
	**45–54**	44 (26%)	36 (22%)	
	**55–64**	23 (13%)	20 (12%)	
	**65+**	7 (4%)	10 (6%)	
**Sex**				0.9
	**Male**	131 (76%)	124 (75%)	
	**Female**	42 (24%)	41 (25%)	
**Employment**				0.06
	**Retired**	29 (17%)	45 (27%)	
	**Unemployed**	91 (53%)	80 (49%)	
	**Employed**	53 (10%)	40 (24%)	
**HIV Status**				0.08
	**Pos**	18 (10%)	8 (5%)	
	**Neg**	155 (90%)	154 (93%)	
	**Missing**	0	3 (2%)	
**Health Care Worker**				
	**Yes**	3 (2%)	5 (3%)	0.4
	**No**	170 (98%)	160 (97%)	
**Police Officer**				
	**Yes**	2 (1%)	3 (2%)	0.6
	**No**	171 (99%)	162 (98%)	
**Contact**				
	**Yes**	42 (24%)	59 (36%)	0.01
	**No**	124 (72%)	105 (63%)	
	**Missing**	7 (4%)	1 (1%)	
**Previous TB history**				
	**Yes**	157 (90.7%)	131 (79.4%)	0.003
	**No**	16 (9.3%)	34 (20.6%)	
**Previously Imprisoned**				
	**Yes**	47 (27%)	39 (24%)	0.3
	**No**	126 (73%)	124 (75%)	
	**Missing**	0	2 (1%)	
**Homeless**				
	**Yes**	8 (5%)	5 (3%)	0.4
	**No**	165 (95%)	159 (96%)	
	**Missing**	0	1 (1%)	
**Alcohol**				
	**Yes**	31 (18%)	31 (19%)	0.8
	**No**	142 (82%)	134 (81%)	
**IDU**				
	**Yes**	13 (7%)	8 (5%)	0.5
	**No**	159 (92%)	155 (94%)	
	**Missing**	1 (1%)	2 (1%)	
**Smoke now**				
	**Yes**	136 (79%)	128 (78%)	0.8
	**No**	37 (21%)	37 (22%)	
**Smoke ever**				
	**Yes**	149 (86%)	139 (84%)	0.3
	**No**	22 (13%)	26 (16%)	
	**Missing**	2 (1%)	0	
**Cavity**				0.6
	**No**	98 (56%)	100 (60%)	
	**Yes, 1 side**	52 (30%)	49 (30%)	
	**Yes, both sides**	22 (13%)	16 (10%)	
	**Missing**	1 (1%)	0	
**TB presentation**				0.2
	**Pulmonary**	165 (95%)	149 (90%)	
	**Extra-pulmonary**	1 (0.5%)	5 (3%)	
	**Both**	6 (4%)	10 (6%)	
	**Missing**	1 (0.5%)	1 (1%)	
**Smear**				0.01
	**Pos**	117 (68%)	89 (54%)	
	**Neg**	56 (32%)	76 (46%)	
**RIF-resistance**				0.6
	**Yes**	28 (16%)	30 (18%)	
	**No**	145 (84%)	135 (82%)	

* Chi-square *p*-values

**Table 2:** Resistance to additional drugs among RIF-resistant TB patients

Characteristics		Pre-Xpert ***N*** = 28	Post-Xpert ***N*** = 30	p *
**Resistance to any additional FLD**				0.05
	**Yes**	28 (100%)	18 (60%)	
	**No**	0	4 (13%)	
	**Missing**	0	8 (27%)	
**Resistance to 4 FLD**				0.02
	**Yes**	21 (75%%)	8 (27%%)	
	**No**	7 (25%)	14 (46%)	
	**Missing**	0	8 (27%)	
**Resistance to any SLD**				0.8
	**Yes**	13 (46%)	10 (33%)	
	**No**	15 (54%)	12 (40%)	
	**Missing**	0	8 (27%)	

* Chi-square *p*-values

Majority of the enrolled patients had positive smear (61%, 206/338) and did not have previous history of TB (85%, 288/338), including 86% (50/58) with positive smear and 74% (43/58) without previous TB history among patients with RIF-resistance.

All RIF-resistant isolates with conventional DST results were also isoniazid-resistant. Among 50 (86%) isolates with DST results, 46 (92%) had resistance to at list one additional first-line drug (FLD); 29 (58%) had resistance to all 4 FLD and 23 (46%) were resistant to at least 1 s-line drug. The *M. tuberculosis* isolates from 4/50 (8%) patients with MDR-TB were resistant to both second-line injectable drug and a fluoroquinolone, and therefore had extensively drug-resistant TB (XDR-TB).

### Time to and predictors of treatment initiation

Among the 58 patients with RIF-resistant TB, time to SLD treatment initiation was missing for four patients from post-Xpert group. One more patient from post-Xpert group did not meet inclusion criteria for survival analysis and was excluded.

We compared time to SLD treatment initiation between 28 RIF-resistant TB patients from pre- and 25 patients from post-Xpert groups. Five patients from pre-Xpert group never started SLD treatment (4 died, 1 lost to follow-up). Among 4 patients who died during TB treatment, 2 had XDR-TB, 1 had pre-XDR TB and 1 patient had resistance to all 1st line drugs. These 4 patients started 1st line TB treatment; their DST results became available after the patients died. The time to death varied between 3 and 33 days since 1st line treatment started. The lost to follow-up patient stopped treatment in 21 days after 1st line treatment initiation; this patient never started SLD. The above 5 patients were censored at the analysis time point.

**Fig. 2:**
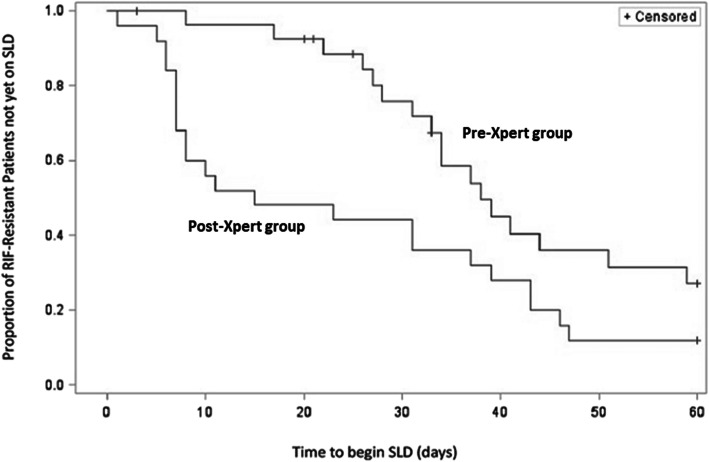
Graph: Time to initiation of treatment with Second Line anti-TB drugs among patients with RIF-resistant TB. Note: SLD treatment regimens of 2 XDR-TB patients included in the graph were adjusted according to SLD DST results

We found that significantly higher proportion of RIF-resistant TB patients started SLD treatment within 1 month of diagnosis in the post-Xpert group compared to the pre-Xpert group (54% vs. 21%, Chi square *p* = 0.01). Within 60 days of initial diagnosis, 39 (74%) of RIF-resistant TB patients started SLD treatment, including 17 in pre- and 22 in post-Xpert groups (61% vs. 88%; Chi square *p* = 0.02). Patients in the post-Xpert group started SLD treatment significantly earlier than those in pre-Xpert group (median 11 vs. 37 days, Log-rank p = 0.02) (Fig. 2). The probability of SLD treatment initiation was significantly higher after Xpert became available in the facility compared to the period before Xpert implementation (aHR:2.06; 95%CI:1.09, 3.89) after controlling for previous imprisonment and alcohol abuse.

There were 145 RIF-susceptible TB patients in the pre-Xpert group and 135 in the post-Xpert group. Median time to initiating treatment decreased but did not significantly differ between RIF-susceptible TB cases diagnosed in the pre-Xpert and post-Xpert groups (3 vs. 2 days, Log-rank *p* = 0.73).

### Impact of Xpert and other factors, associated with treatment outcome

We analyzed impact of Xpert and other factors that could be associated with treatment outcomes for RIF-resistant and RIF-susceptible TB patients. From 58 RIF-resistant TB patients 5 did not have data on treatment outcome. Among the 53/58 RIF-resistant TB patients with available treatment outcome, the median age was 38 years old (IQR: 31–50), majority (83%) were male, 6 (12%) were HIV positive, and 21 (41%) had contact with a known TB case. Of these 53 patients, 28 (53%) had successful outcomes and 25 had poor outcomes. Positive HIV status, smoking history, unemployment, and history of imprisonment significantly associated with poor treatment outcome (Table 3). Availability of Xpert for diagnosis of TB patients accessing care in the Dispensary in 2012 was not associated with treatment outcome (OR:0.69; 95%CI:0.23,2.06) in patients with MDR-TB.

**Table 3:** Factors associated with treatment outcome among RIF-susceptible and RIF-resistant patients

		RIF-susceptible Patients (252)				RIF-resistant Patients (53)			
		Poor outcome	Successful outcome	OR (95% CI)*	p *	Poor outcome	Successful outcome	OR (95% CI)*	p*
		*N* = 53	*N* = 199			*N* = 25	*N* = 28		
**Age**	**15–24**	4	28	0.49 (0.13, 1.56)	0.25	3	2	2.56 (0.29, 27.91)	0.41
	**25–34**	16	55	ref		5	9	ref	
	**35–44**	11	39	0.97 (0.39, 2.33)	0.95	8	7	2.01 (0.44, 9.70)	0.37
	**45–54**	13	44	1.02 (0.43, 2.35)	0.97	6	7	1.52 (0.31, 7.69)	0.61
	**55–64**	8	22	1.25 (0.45, 3.33)	0.66	2	2	1.74 (0.14, 21.3)	0.65
	**65+**	1	11	0.32 (0.01, 2.07)	0.29	1	1	1.73 (0.04, 77.51)	0.75
**Sex**	**Male**	45	141	2.31 (1.05, 5.53)	0.04	23	21	3.74 (0.74, 28.83)	0.11
	**Female**	8	58	ref		2	7	ref	
**Employment**	**Retired**	10	39	3.01 (1.01, 9.54)	0.05	4	10	1.57 (0.22, 14.97)	0.68
	**Unemployed**	37	89	4.89 (2.03, 13.38)	< 0.01	19	10	7.19 (1.37, 57.69)	0.02
	**Employed**	6	71	ref		2	8	ref	
HIV Positive **	**Yes**	8	9	3.71 (1.31, 10.4)	0.01	6	0	**–**	< 0.01
	**No**	45	189	ref		19	27		
**Health Care Worker**	**Yes**	2	4	1.91 (0.24, 11.04)	0.48	0	1	**–**	0.52
	**No**	51	195	ref		25	27		
**Police Officer**	**Yes**	0	4	**–**	0.39	0	1	**–**	0.52
	**No**	53	195	ref		25	27		
Contact ***	**Yes**	13	57	0.84 (0.40, 1.67)	0.63	13	8	3.17 (0.99, 10.68)	0.05
	**No**	38	139	ref		10	20	ref	
**Previous TB history**	**Yes**	12	19	2.77 (1.24, 6.16)	<.0001	6	7	0.95 (0.27, 3.32)	0.9
	**No**	41	180	ref		19	21	ref	
**Previously Imprisoned**^**#**^	**Yes**	19	40	2.19 (1.12, 4.23)	0.02	12	5	4.12 (1.20, 15.7)	0.02
	**No**	34	157	ref		13	23	ref	
**Homeless**^**##**^	**Yes**	4	2	7.91 (1.37, 63.3)	0.02	2	2	1.13 (0.11, 11.57)	0.91
	**No**	49	196	ref		23	26	ref	
**Alcohol**	**Yes**	12	27	1.86 (0.84, 3.96)	0.12	8	8	1.17 (0.35, 3.92)	0.79
	**No**	41	172	ref		17	20	ref	
**IDU**^**###**^	**Yes**	4	8	1.96 (0.50, 6.78)	0.31	5	1	6.86 (0.87, 175)	0.07
	**No**	49	189	ref		19	27	ref	
**Smoke now**	**Yes**	46	146	2.38 (1.05, 6.02)	0.04	23	22	3.07 (0.58, 24.16)	0.20
	**No**	7	53	ref		2	6	ref	
**Smoke ever**^**$**^	**Yes**	49	162	3.62 (1.17, 15.38)	0.02	25	23	**–**	0.03
	**No**	3	36	ref		0	5		
**Xpert**	**Post-Xpert**	20	94	0.68 (0.36, 1.26)	0.22	11	15	0.69 (0.23, 2.06)	0.50
	**Pre-Xpert**	33	105	ref		14	13	ref	
**Cavity**^**$$**^	**Yes**	29	65	2.6 (1.4, 4.8)	0.003	18	15	2.2 (0.7, 7.0)	0.17
	**No**	23	134	ref		7	13	ref	
**TB presentation**^**$$$**^	**Pulmonary**	47	184	0.96 (0.3, 3.0)	0.9	23	28	**–**	0.13
	**Any EPT**	4	15	ref		2	0		
**Smear**	**Pos**	37	105	2.1 (1.1, 3.9)	0.03	24	22	6.5 (0.7, 58.7)	0.09
	**Neg**	16	94	ref		1	6	ref	
**Any additional res to FLD**^**&**^	**Yes**	N/A	N/A	N/A	N/A	23	19	3.6 (0.3, 37.8)	0.3
	**No**					1	3	ref	
**Resistance to 4 FLD**^**&&**^	**Yes**	N/A	N/A	N/A	N/A	16	11	2.0 (0.6, 6.6)	0.3
	**No**					8	11	ref	
**Any SLD**^**&&&**^	**Yes**	N/A	N/A	N/A	N/A	8	13	0.4 (0.1, 1.5)	0.08
	**No**					16	9	ref	

* Conditional maximum likelihood estimate odds ratios and corresponding mid-exact p-values** HIV status was missing for 2 patients (1 RIF-susceptible; 1 RIF-resistant)*** Contact info was missing for 7 patients (5 RIF-susceptible; 2 RIF-resistant)^#^ Prison history and smoking history were missing for 2 patients (RIF-susceptible)^##^ Homeless status was missing for 1 patient (RIF-susceptible)^###^ IDU history was missing for 3 patients (2 RIF-susceptible; 1 RIF-resistant)

^$^ Smoke ever was missing for 2 patients (RIF-susceptible)

^$$^ Cavity information was missing for 1 patient (RIF-susceptible)

^$$$^ TB presentation was missing for 2 patients (RIF-susceptible)

^&^ Any additional resistance to first-line drugs (FLD) was missing for 7 patients (RIF-resistant)

^&&^ Resistance to 4 FLD was missing for 7 patients (RIF-resistant)

^&&&^ Resistance to any second-line drugs (SLD) was missing for 7 patients (RIF-resistant)

Among 280 RIF-susceptible patients, treatment outcomes were available for 138 patients in the pre-Xpert group and for 114 patients in the post-Xpert group. The median age of 252/280 RIF-susceptible TB cases with treatment outcome was 38 years old (IQR: 29–51), most were males (74%), 17 (7%) were HIV positive, and 28% had contact with a known TB case. Overall, 199 (79%) RIF-susceptible cases had successful outcome and 53 (21%) had poor outcome. Poor treatment outcome among RIF-susceptible TB patients was associated with male gender, history of imprisonment, homelessness, unemployment, having ever smoked, positive HIV status, previous history of TB, positive smear and presence of cavities on X-ray at the start of treatment (Table 3). Availability of Xpert for diagnosis of RIF-susceptible TB patients accessing care in the Dispensary in 2012 was not associated with treatment outcome (OR:0.68; 95%CI:0.36,1.26).

## Discussion

RIF-resistant TB was present in 17% of TB patients enrolled in the study in the Vladimir TB Dispensary, similar to WHO MDR-TB prevalence estimates for Russia [1]. We found that availability of Xpert for diagnosis significantly reduced the median time to SLD treatment for RIF-resistant patients from 37 to 11 days, despite being in the initial stage of implementation of a new technology in the facility. Similar results have been reported by researchers in Zimbabwe, India, South Africa, Latvia and Korea [4, 13–17]. Successful outcomes were experienced by 79% RIF-susceptible patients, and 53% of RIF-resistant patients. Availability of Xpert for initial diagnosis was not associated with improved treatment outcomes, consistent with findings of clinical studies in South Africa and Korea [13, 17].

Our results of the analysis of clinical factors associated with treatment outcome among RIF-susceptible TB patients (HIV infection, smear positivity and cavitary disease) are in line with the findings from the recent patient-level pooled analysis of treatment-shortening regimens for drug-susceptible pulmonary tuberculosis [18]*.*

There were several limitations to the study. Due to unforeseen political circumstances we were unable to make the full sample size which limited statistical power of the study. However, this fact did not diminish the importance of our finding about the significant reduction of the time to SLD treatment for RIF-resistant patients. There is also a possibility that the treatment regimen for some patients was incompletely recorded. We used the most complete and accurate data for each patient, and excluded patients with incomplete treatment information. Data were collected at the initial stage of implementation of Xpert in the facility, thus the new diagnostic algorithm was still under development, including logistics, staff training and information exchange. This assessment would be more accurate if conducted after full implementation of the technology. However, this is major strength of the study, as it provides data on the real-time implementation of Xpert in a clinical setting in Vladimir Region, Russia.

## Conclusion

In our study we observed that availability of Xpert for initial diagnosis significantly reduced the time to SLD treatment for RIF-resistant patients in the Vladimir TB Dispensary. Although implementation of rapid diagnostics did not improve treatment outcomes, early diagnosis of drug-resistant TB is important for selection of treatment regimen and initiation of the appropriate therapy more rapidly, which can further reduce the likelihood of transmission of drug-resistant strains of TB in the community.

### Disclaimer

The findings and conclusions in this manuscript are those of the authors and do not necessarily represent the official position of the U.S. Centers for Disease Control and Prevention.

## Data Availability

The dataset generated and analyzed during the current study is not publicly available due luck of resources required to prepare the dataset for public use but can be available from the corresponding author on reasonable request.

## Abbreviations

aHR: Adjusted Hazard Ratio
CI: Confidence Intervals
DNA: Deoxyribonucleic acid
DS TB: Drug-susceptible TB
DST: Drug Susceptibility Testing
HR: Hazard Ratio
FLD: First-line anti-TB drugs
IQR: Interquartile Range
LJ: Lowënstein-Jensen
MDR: Multi-Drug Resistance
MDR-TB: Multi-Drug Resistant Tuberculosis
M.tuberculosis: *Mycobacterium tuberculosis*
OR: Odds Ratio
RIF: Rifampicin
SLD: Second-line anti-TB drugs
TB: Tuberculosis
WHO: World Health Organization
XDR TB: Extensively drug-resistant TB
Xpert: GeneXpert MTB/RIF**®**
